# Predicting dairy cattle PL via longitudinal rumen microbiome dynamics using machine learning approaches

**DOI:** 10.1128/spectrum.02969-25

**Published:** 2026-04-08

**Authors:** Panping Yang, Zhuoxuan Wu, Sixi Zhang, Lei Yan, Yuefei Yan, Feng Zhou, Xiaoli Ren, Yujia Li, Shuhua Gu, Shanshan Gao, Zhiyang Zhang, Yang Zhang, Huiyuan Zhu, Huixia Li, Zhen Zhang

**Affiliations:** 1 College of Animal Science and Technology, Nanjing Agricultural University 70578, Nanjing, Jiangsu, China; 2 Henan Dairy Herd Improvement Center, Zhengzhou, Henan, China; 3 Runan County Product Quality Inspection and Testing Center, Zhumadian, Henan, China; 4 Henan Provincial International Joint Laboratory for Dairy Health Farming, Zhengzhou, Henan, China; 5 Henan Seed Industry Development Center, Zhengzhou, Hennan, China; 6 Henan Dairy Production Performance Measurement Co., Ltd., Zhengzhou, Henan, China; 7 Henan Breeding and Poultry Industry Research Institute Co., Ltd., Zhengzhou, Henan, China; Cleveland Clinic Lerner Research Institute, Cleveland, Ohio, USA

**Keywords:** rumen microbiota, dairy cow, parity, machine learning, biomarker

## Abstract

Aging is a spontaneous biological process involving intricate regulatory mechanisms over time. Studies in mice and humans indicate that the gut microbiota is closely linked to the aging process and plays an important role in it. However, the relationship between the rumen microbiota and aging in dairy cows remains unclear. In this study, we characterized rumen microbial differences across different parities in 341 dairy cows using 16S rRNA amplicon sequencing and identified microbial markers associated with productive lifespan (PL) and farm profitability through machine learning analysis. Our findings reveal that as parity increases, the rumen microbiota undergoes systematic succession: alpha diversity indices significantly decrease, microbial interactions weaken, the abundance of *Proteobacteria* increases, while the abundance of *Bacteroidetes* decreases in higher-parity cows. By integrating machine learning with 16S sequencing, we identified characteristic microbial markers predictive of PL and farm profitability. Specifically, the support vector regression model achieved a predictive performance with an area under the curve (AUC) of 0.788 and identified eight key genera associated with the PL of dairy cows. Meanwhile, the random forest (RFTEST) model attained an AUC of 0.763 and selected eight key microorganisms linked to the economic benefits of the farm, with fivefold cross-validation confirming the reliability of RFTEST. Combined with SHapley Additive exPlanations (SHAP) analysis, the genus-level taxa *Eubacterium_hallii_group* and *Prevotella_7* can serve as indicator strains for PL and farm profitability in dairy cows. Therefore, alterations in the rumen microbiota may serve as a key driver of aging in dairy cows. This study aims to provide insights for improving PL and farm profitability through the modulation of rumen microbiota.

**IMPORTANCE:**

In the dairy industry, longevity is a critical economic trait that directly impacts overall farm profitability. Although dairy cows have a natural lifespan of approximately 20 years—with optimal productivity often extending beyond the fifth parity—their average PL is only about 2.7 parities. Identifying factors influencing PL is therefore crucial. Given the vital role of the rumen microbiota in regulating dairy performance, milk fat/protein synthesis, and other key physiological processes, elucidating its correlation with PL is essential for developing probiotic interventions to enhance longevity. Furthermore, early detection of aging-associated microbial signatures could facilitate proactive adjustments to feeding strategies. Notably, this is the first study to link parity-driven microbiome succession with PL prediction in dairy cattle. Consequently, by identifying microbial molecular markers linked to PL and potential probiotic targets, this study highlights promising opportunities to improve dairy cow health and advance sustainable dairy farming practices.

## INTRODUCTION

Productive longevity represents a critical economic indicator in dairy farming, directly determining herd profitability (1). Extending dairy cow longevity significantly reduces replacement heifer rearing costs and optimizes resource allocation. Defined as the interval from first calving to culling due to declining productivity or health issues, global productive longevity exhibits substantial disparities: exceeding 4.5 years in developed livestock systems (EU/US) versus ~2.5 parities in Chinese Holsteins (2, 3). Compared to cows' ~20-year natural lifespan, this premature culling prevents attainment of peak productivity (typically ≥5th parity), resulting in significant economic potential wastage. Consequently, elucidating longevity regulatory mechanisms has become an international research priority in animal science due to its profound economic implications. Conventional research attributes dairy longevity to genetic background, epigenetic regulation, and environmental factors (climate, management), overlooking symbiotic microbiota’s contributions (4). Human and murine studies demonstrate age-dependent declines in mucin-metabolizing taxa (*Clostridiaceae*, *Akkermansiaceae*, and *Bifidobacteriaceae*, *Bacteroidaceae*), causing intestinal barrier thinning, microbial-epithelial contact, and inflammatory cascades (5, 6). Low-grade chronic inflammation—a hallmark of aging—stems from geriatric gut microbiome alterations that perturb host metabolism (7). As postulated by Nobel laureate Élie Metchnikoff (1907), microbial metabolite toxicity drives aging, suggesting lifespan extension via microbiota modulation (8). Within ruminants' forestomach, the rumen harbors extraordinarily complex microbial consortia (10^10^–10^11^ cells/mL) that regulate milk synthesis and disease resistance.

Within the forestomach of ruminants, the rumen harbors an extraordinarily complex microbial consortium (10¹⁰–10¹¹ cells/mL) that is closely associated with host growth rate, body weight gain, feed efficiency, milk synthesis, and other production traits. For instance, *Streptococcus*, *Candidatus Saccharimonans*, and *Succinivibrionaceae* UCG-001 are closely linked to growth rate in goats (9). *Selenomonas ruminantium*, *Selenomonas* sp., and *Faecalibacterium prausnitzii* enhance feed digestibility by producing cellulolytic enzymes and propionate-generating enzymes (10). *Prevotella* may improve dairy production performance by modulating amino acid metabolism and carbohydrate breakdown (11). Furthermore, rumen dysbiosis triggers mammary inflammation via the “gut-mammary axis,” thereby inducing host mastitis (12), yet their role in longevity regulation remains underexplored.

To address this gap, we hypothesize that rumen microbiota fundamentally drives dairy cow aging. We profiled rumen microbiomes from 341 cows across parities, analyzed structural and compositional dynamics to characterize parity-associated microbiome succession, systematically evaluated feature microorganisms to identify predictive microbial biomarkers, and constructed machine learning models for PL and profitability. We selected 8 regression algorithms and 12 classification methods for longevity/production efficiency prediction. This framework provides theoretical foundations for precision microbiome-directed feeding strategies to enhance dairy longevity.

## MATERIALS AND METHODS

### Animal handling and experimental diets

To investigate the relationship between rumen microbiota and PL in dairy cows across different parities, this study selected 341 cows of varying parities scheduled for culling to conduct the experiment, with data collection completed by July 1, 2024. Prior to rumen fluid collection, all cattle were managed under standardized farm protocols. Rumen fluid samples were collected from each cow by a professional animal husbandry specialist via oral stomach tube and subsequently filtered through quadruple-layer sterile gauze. A 5 mL aliquot was flash-frozen in liquid nitrogen and stored at −80°C for microbial analysis.

### Microbial diversity sequencing and analysis

Total microbial genomic DNA was extracted from all rumen fluid samples using the E.Z.N.A. Soil DNA Kit (Omega Bio-tek, USA) per manufacturer’s instructions. DNA quality and concentration were verified via 1.0% agarose gel electrophoresis and NanoDrop2000 spectrophotometry (Thermo Scientific, USA), with aliquots stored at −80°C. The V3-V4 hypervariable region of bacterial 16S rRNA genes was amplified with primer pair 338F (5′-ACTCCTACGGGAGGCAGCAG-3′) and 806R (5′-GGACTACHVGGGTWTCTAAT-3′) (13) using a T100 Thermal Cycler (Bio-Rad, USA). PCR reactions (20 μL) contained 4 μL 5× Fast Pfu Buffer, 2 μL 2.5 mM dNTPs, 0.8 μL each primer (5 μM), 0.4 μL Fast Pfu Polymerase, 10 ng template DNA, and ddH_2_O to volume. Cycling conditions were as follows: 95°C for 3 min; 27 cycles of 95°C/30 s, 55°C/30 s, 72°C/45 s; 72°C for 10 min; hold at 4°C. Amplicons were electrophoresed on 2% agarose gels, purified with a PCR Clean-Up Kit (Yuhua, China), and quantified by Qubit 4.0 (Thermo Fisher, USA). Equimolar purified amplicons were pooled and sequenced (2 × 250 bp) on Illumina NextSeq 2000 (Illumina, USA) by Majorbio (Shanghai). Raw data were deposited in NCBI SRA (PRJNA1375500).

### Bioinformatic workflow

Demultiplexed raw FASTQ files were processed through (i) quality control via fastp; (ii) FLASH assembly (discarding reads <50 bp or average quality <20; merging only with >10 bp overlap); (iii) sample separation by barcode/primer; and (iv) UPARSE 7.1 OTU clustering at 97% similarity after chloroplast removal. Sequences were rarefied to 20,000 reads/sample (Good’s coverage: 99.09%). Alpha diversity (Chao1, ACE) and rarefaction curves were calculated in Mothur v1.30.1. Combining production performance data with microbial diversity, we established a relationship model between Chao1 richness and milk yield. Our hypothesis was that microbial diversity correlates with milk yield. The model is represented by *Y* = β0 + β1X1 + β2X2 + … + βnXn + *ε*, where *Y* denotes the dependent variable, X1, X2, …, Xn denote the independent variables, β0, β1, …, βn are the model coefficients, and *ε* is the error term. We obtained the optimal model coefficients using the least squares method. The distribution frequency of the dependent variable (Chao1 richness) and the independent variable (milk yield) was statistically analyzed using a boundary histogram. The Spearman correlation coefficient was used to measure the diversity correlation between milk yield and microbial diversity. Based on the sequencing results of diversity, principal component analysis (PCA) of different group communities was performed using the R package ggbiplot (GitHub—vqv/ggbiplot: a biplot based on ggplot2). Differential biomarkers were identified by Kruskal–Wallis test with LDA effect size quantification. To analyze microbial interactions across different groups, we performed microbial co-occurrence network analysis using the R package WGCNA, retaining microorganisms with Pearson correlation coefficients >0.8 for network construction.

### Functional prediction

Functional pathway analysis was performed using PICRUSt2 software based on the Kyoto Encyclopedia of Genes and Genomes (KEGG) and Clusters of Orthologous Groups (COG) databases, with MinPath used to identify gene pathways. First, amplicon sequence variants (ASV) sequences from different groups were aligned with internal reference sequences, and the ASVs were placed into the corresponding reference tree. This process enabled inference of gene family copy numbers for each ASV, prediction of the gene content of each ASV, and determination of gene family abundances in each sample. Subsequently, gene family information was aligned with the corresponding functional databases (KEGG, COG) to obtain the corresponding functional information and abundance data for each sample.

### Analysis of rumen microbial community targets associated with dairy cow PL using machine learning algorithms

To determine the optimal microbial prevalence threshold for predicting PL, we designed a sensitivity analysis workflow. First, microbial prevalence thresholds were set within the observed range from the minimum (0.001%) to maximum (100%) values, generating a series of candidate thresholds at 0.5% intervals. For each candidate threshold, subsets of microbes with prevalence greater than or equal to the threshold were filtered as features. Subsequently, a multiple linear regression model was constructed with PL as the dependent variable and the relative abundances of the filtered microbes as independent variables. To avoid overfitting and multicollinearity issues caused by high-dimensional data, only the top 3 most abundant microbes were retained in the model when the number of features exceeded 3. The model formula is as follows:


PL∼Microbe1+Microbe2+Microbe3

For each fitted model, we calculated the coefficient of determination (*R*²) and root mean square error (RMSE) to evaluate the predictive performance under different thresholds. Finally, the prevalence threshold that maximized the *R*² value was selected as the optimal threshold. To verify the robustness of the optimal threshold, the original data set was randomly split into a training set (70%) and a test set (30%). The above sensitivity analysis was performed exclusively on the training set to determine the optimal threshold. This threshold was then applied to the independent test set, and the model’s *R*² and RMSE on the test set were calculated to assess its generalization ability. To further evaluate the model’s classification performance, continuous PL was converted into a binary variable based on the herd average PL(>2.59 = 1, <2.59 = 0). Using microbial features filtered by the optimal prevalence threshold, a logistic regression model was constructed to predict the binary outcomes. The receiver operating characteristic (ROC) curve was plotted, and the AUC with its 95% confidence interval was calculated to quantify the discriminative efficacy of the model. Additionally, the optimal classification threshold for logistic regression prediction probability was determined using Youden’s index, and corresponding metrics, including sensitivity, specificity, and accuracy, were calculated.

To construct a predictive model for dairy cows' PL based on rumen microbiota, we first performed Boruta feature selection using the R package Boruta (parameters: maxRuns = 100, random seed set to 123, trace information doTrace = 2) and LASSO (Least Absolute Shrinkage and Selection Operator) regression feature selection using the R package glmnet. The Boruta algorithm is a technique for identifying key features in a data set. Its core principle involves evaluating feature importance by comparing the *Z*-value of each feature with the *Z*-value of its corresponding “shadow feature.” Specifically, the algorithm first copies all real features and performs a random permutation, then calculates the *Z*-values of each feature using a random forest (RFTEST) model; simultaneously, the *Z*-values of “shadow features” are generated by randomly shuffling the real features (14). When the *Z*-value of a real feature significantly exceeds the maximum *Z*-value of shadow features in multiple independent experiments, the feature is classified as “important” (green zone), namely “acceptable variable”; conversely, it is labeled as “non-important” (red zone), namely “unacceptable variable.” Acceptable variables are retained during the feature selection process and are considered to make a positive contribution to model performance; unacceptable variables are ultimately eliminated from the feature set by the algorithm because they fail to demonstrate predictive power for the target variable during the selection phase. In addition, the Boruta algorithm is also used to explore the degree of importance of stress hyperglycemia ratio as a predictor variable (15). LASSO regression is a linear regression method that employs shrinkage techniques, with its core mechanism involving the shrinkage of data values toward a central point (e.g., the mean) for model optimization. This regression approach implements L1 regularization, a process that introduces a penalty term equal to the absolute magnitude of the coefficients. This regularization strategy facilitates the construction of parsimonious and sparse model structures, such as those with a reduced number of parameters (16). We combined the overlapping microbes identified by Boruta and LASSO regression analyses as key variables for predictive model construction and evaluation. Following traditional machine learning methods, we divided the data set into a training set and a validation set at an 8:2 ratio. Using Python 3.10.6, we built seven regression models, including Adaptive Boosting (AdaBoostRTEST), Categorical Boosting (CatBoostRTEST), Decision Tree, (DecisionTreeRTEST), Light Gradient Boosting Machine (LGBMRTEST), Support Vector Regression (SVRTEST), eXtreme Gradient Boosting (XGBRTEST), Random Forest Regression (RFRTEST), conducting individual analysis on the screened characteristic microorganisms to construct microbiota-based predictive models for dairy cows' PL. Hyperparameter optimization was conducted during the development of machine learning models. The training and validation sets were employed for model construction and evaluation, respectively. ROC curves with their associated AUC were applied to assess model performance, while decision curve analysis (DCA) was utilized to evaluate clinical utility. Meanwhile, calibration curves were leveraged to validate the accuracy of absolute risk predictions generated by the models.

### Machine learning algorithm-based analysis of associations between microbial communities and ranch economic returns

**Fig 1:**
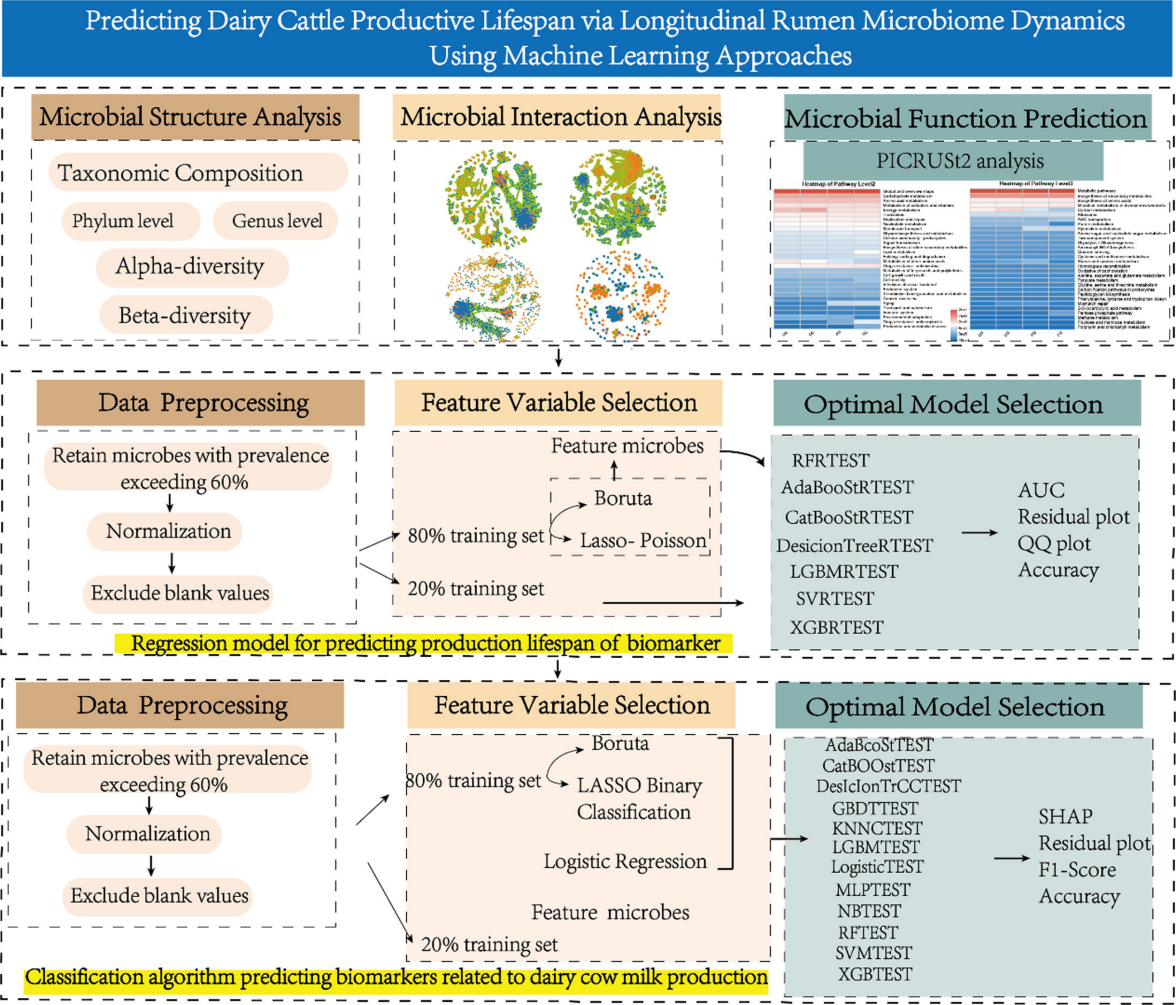
Summary of materials and methods.

Dairy cow parity serves as a critical reference indicator for ranch economic returns. By integrating the economic input and output data of individual cows, we established 2.59 parities as the threshold, delineating those exceeding 2.59 parities as the profitable cohort and those below as the unprofitable cohort. We used Boruta and LASSO binomial feature selection to identify common feature microbes (Fig. 1), which were then used as feature variables for subsequent predictive models. Using Python 3.10.6, we constructed 12 classification prediction models (AdaBoostTEST, CatBoostTEST, DecisionTreeTEST, GBDTTEST, KNNCTEST, LGBMTEST, LogisticTEST, MLPTEST, NBTEST, RFTEST, SVMTEST, and XGBTEST). Model optimization and robustness validation were performed as described above.

To better evaluate the optimal model and prevent overfitting, fivefold cross-validation was performed. The training set was randomly divided into five mutually exclusive subsets; in each iteration, four subsets were used as the training set and the remaining one subset as the validation set. This process was repeated five times. The reliability of the model was comprehensively assessed using ROC curves with AUC values, and precision-recall curves. This process was implemented using Python 3.10.6 with the *sklearn.metrics module*, *sklearn.metrics*, and the *matplotlib* package (17).

## RESULTS

### Production performance and microbiome composition across parities

This study initially performed a large-scale population screening and observed that peak-lactation (31–100 d) dairy cows exhibited significant variations in milk yield. Specifically, milk production demonstrated a declining trend as parity increased (Fig. S1A and B). Statistical analysis of the productive performance of 341 selected dairy cows across different parities revealed regular patterns of variation in milk fat, milk protein, and milk yield. Specifically, milk yield exhibited a decreasing trend with increasing parity, whereas milk protein content showed an upward trend (Table S1). This phenomenon may be attributed to a concentration effect resulting from the decreased milk yield in older, higher-parity cows. With increasing parity, these cows likely allocate more energy toward maintaining their own metabolic functions—such as tissue repair and disease resistance—while their capacity for milk fat secretion diminishes. When energy intake remains relatively stable, the proportion of nutrients directed to the mammary gland decreases. However, since the synthesis efficiency of milk fat and protein remains relatively consistent, this leads to higher concentrations of fat and protein in their milk.

**Fig 2:**
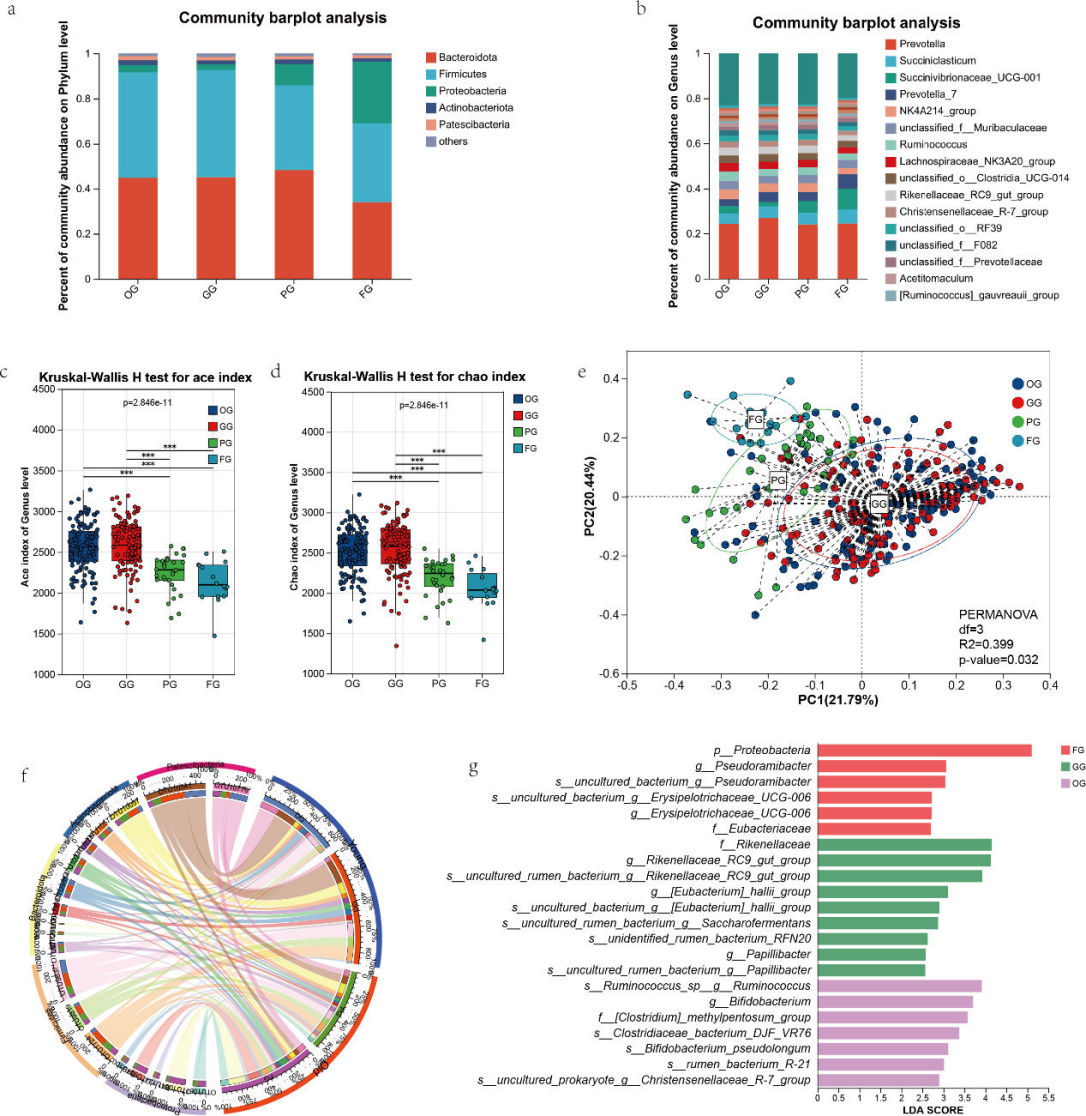
Statistical analysis of microbial community structure. OG: primiparous cows (first lactation), GG: multiparous cows (second/third lactation), PG: multiparous cows (fourth lactation), FG: multiparous cows (fifth lactation). (**a**) Phylum-level microbial composition across groups. (**b**) Genus-level microbial composition. (**c**) Microbial alpha diversity (ACE index) analyzed by Kruskal–Wallis H test. (**d**) Microbial alpha diversity (Chao1 index) analyzed by Kruskal–Wallis H test. (**e**) Principal coordinate analysis of rumen microbiota based on unweighted UniFrac distance. Principal components 1 and 2 (PC1/PC2) explain 21.79% and 20.44% of variation, respectively. (**f**) Chord diagram depicting rumen microbial interactions between young (OG, GG) and aged (PG, FG) cow groups. (**g**) LEfSe identifying differentially abundant taxa (LDA score threshold > 2.5).

Based on productive performance, cows were categorized into four groups: OG (1st parity), GG (2nd to 3rd parity), PG (4th parity), and FG (≥5th parity). Analysis of the rumen microbiota composition among dairy cows of different parities under identical feeding management revealed considerable variation in the relative abundances of the phyla *Bacteroidetes* and *Proteobacteria* across parity groups. The relative abundance of *Firmicutes* showed no significant differences among the groups. Specifically, the abundance of *Proteobacteria* increased significantly in the PG and FG groups, while *Bacteroidetes* exhibited an initial rise followed by a decline with advancing parity (Fig. 2a). At the genus level, the abundances of *Succinivibrionaceae_UCG_001* and *Prevotella-7* also increased with higher parity (Fig. 2b). Using the ACE and Chao indices to evaluate rumen microbial diversity, alpha diversity was found to decrease with increasing parity, which may be related to the physiological stage of high-parity dairy cows, which have experienced multiple pregnancies and lactation stress, often accompanied by low energy utilization efficiency, negative energy balance, and risk of metabolic disorders. This may be associated with dysbiosis of dominant microbiota, characterized by decreased abundance of cellulolytic bacteria, *Prevotella*, etc., while acid-tolerant bacteria overproliferate, ultimately reducing microbial diversity. A Kruskal–Wallis H test confirmed significant differences in rumen microbial composition among cows of different parities (Fig. 2c and d). Further PCA of rumen microbial samples highlighted distinct group-specific characteristics: OG and GG groups displayed clear clustering, whereas FG and PG groups were significantly separated. Permutational multivariate analysis of variance indicated that parity grouping had a significant effect on multivariate microbial responses (*R*² = 0.399, df = 3, *P* < 0.05), suggesting parity is a key driver of functional differences in rumen microorganisms (Fig. 2e). Additionally, correlation analysis revealed that the microbial Chao index was negatively correlated with PL but positively correlated with milk yield (Fig. S2). To further validate parity-related microbial abundance differences, cows with 1–3 parities were classified as “young,” and those with ≥4 parities as “old.” A chord diagram visualized microbial abundances between the two groups, showing that *Proteobacteria* and *Actinobacteriota* were closely connected with wider arcs—indicating higher abundances and stronger biological interactions in high-parity cows—whereas *Firmicutes*, *Bacteroidetes*, and *Patescibacteria* were more strongly linked to the “young” group (Fig. 2f). Linear discriminant analysis effect size (LEfSe) was applied to identify differential taxa across groups. Fig. 2g presents taxa with LDA scores >2.5, including *p__Proteobacteria*, *g__Pseudoramibacter*, *g__Erysipelotrichaceae*, and *f__Eubacteriaceae* as significantly enriched in the FG group. Conversely, rumen probiotics such as *f__Rikenellaceae*, *g__Rikenellaceae_RC9_gut_group*, *g__Bifidobacterium*, and *g__Bifidobacterium pseudolongum* were identified in the OG and GG groups (Fig. 2g). Based on the above-described microbial characteristics of dairy cows with different parities, we found that as dairy cow parity increases, rumen microbial composition and diversity both undergo changes. Additionally, the results of this study are consistent with the trends of gut microbial changes during aging in mice and humans, where increased abundance of *Proteobacteria* and decreased alpha diversity may be one of the characteristics of microbial dysbiosis (18, 19).

### Analysis of rumen microbial interactions in dairy cows of different parities

**Fig 3:**
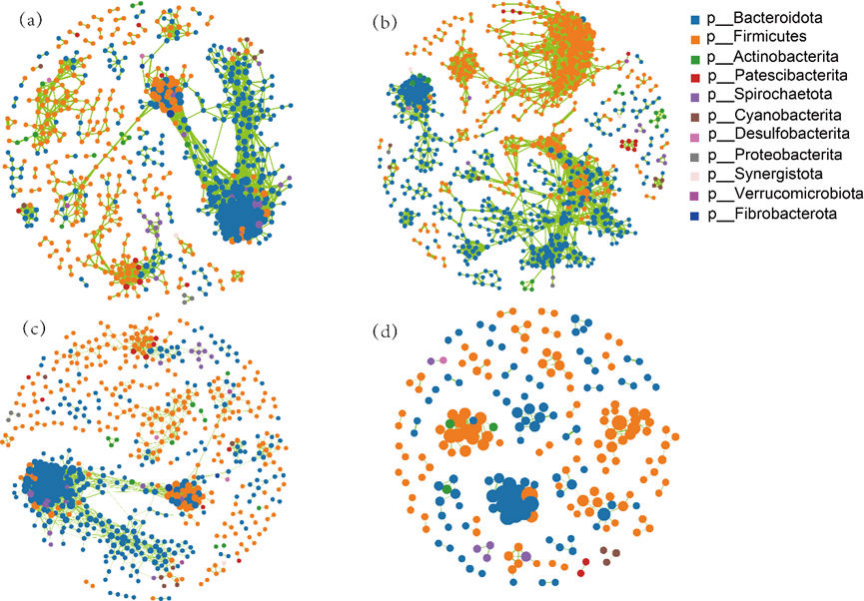
Network interaction analysis of rumen microbiota across different groups. Panels **a to d** represent OG, GG, PG, and FG groups, respectively.

Using molecular ecological network analysis and visualization tools based on 16S rRNA sequencing, interrelationships among gut microorganisms were explored. The rumen microbial interaction networks of OG and GG groups contained 1,363 nodes/6,173 edges and 1,782 nodes/5,803 edges, respectively, whereas those of PG and FG groups included 849 nodes/637 edges and 153 nodes/145 edges. Notably, with increasing parity, negative microbial interactions were enhanced (Table S2), and rumen microbial interactions shifted from “expansion” to “simplification” (Fig. 3).

### Functional prediction based on Phylogenetic Investigation of Communities by Reconstruction of Unobserved States

Phylogenetic Investigation of Communities by Reconstruction of Unobserved States (PICRUSt) is a bioinformatics toolkit for predicting metagenomic functions from marker gene sequencing data. We normalized the operational taxonomic unit) abundance tables across groups using PICRUSt, then mapped Greengenes IDs to KEGG and COG databases to retrieve functional modules and calculate module abundances per sample.

**Fig 4:**
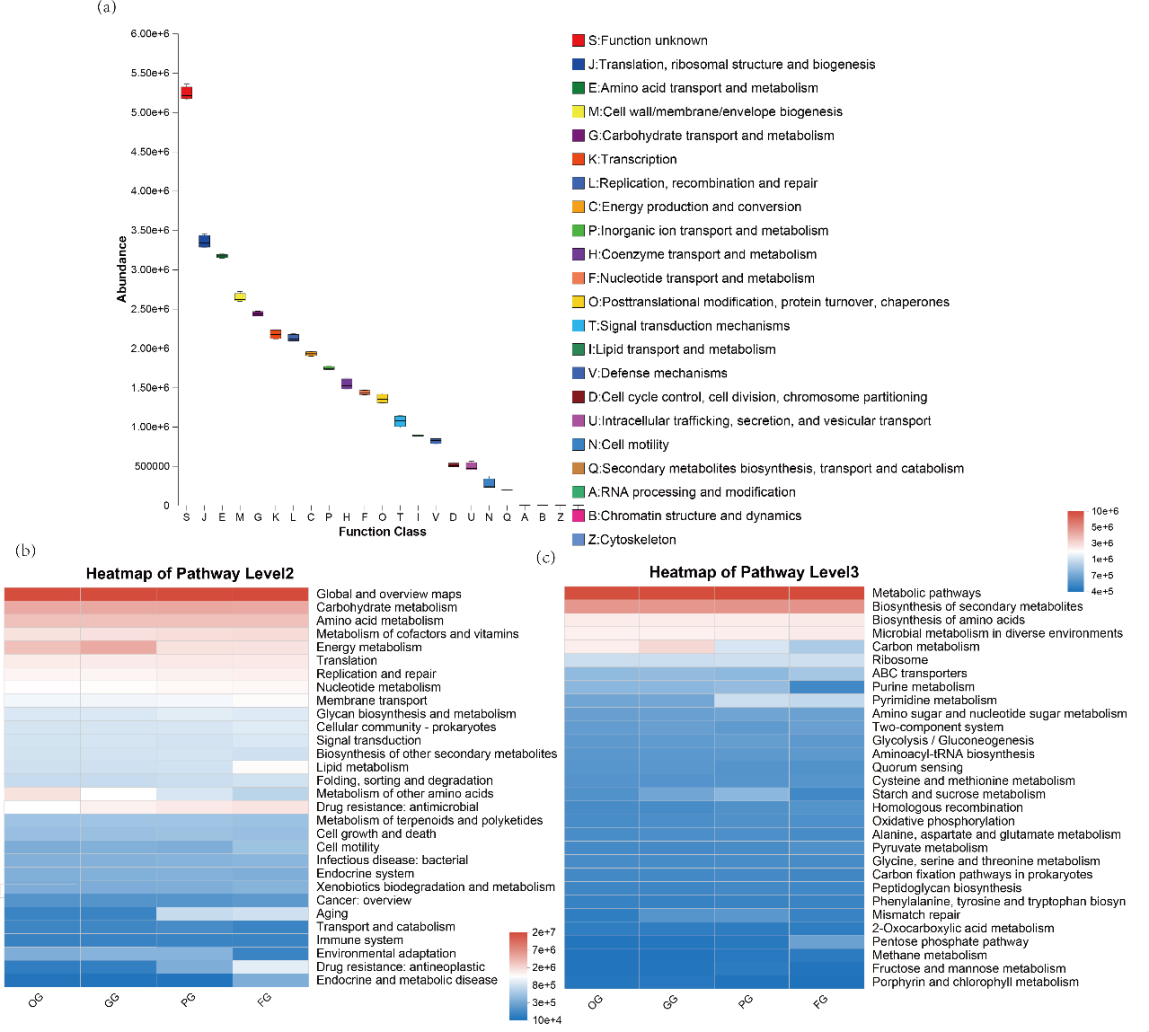
(**a**) Box plots showing abundance distribution of COG categories. (**b**) Heatmap displaying KEGG pathway abundance at level 2 across groups. (**c**) Heatmap of KEGG pathway abundance at level 3 among groups.

PICRUSt2, coupled with the COG database, predicted functional potentials of bacterial microbiota in tissues. Box plots display differential biological functions of microbial communities (Fig. 4a). At pathway level 2, significant variations were observed in energy metabolism, lipid metabolism, amino acid metabolism, and antimicrobial drug resistance (Fig. 4b). Further analysis at level 3 revealed that carbon metabolism was significantly enriched in OG and GG groups, differential enrichment of starch and sucrose metabolism in GG versus PG groups, FG group microbiota showed strong associations with purine metabolism and mismatch repair pathways (Fig. 4c).

### Prediction of rumen microorganisms associated with dairy cows' PL using regression models

**Fig 5:**
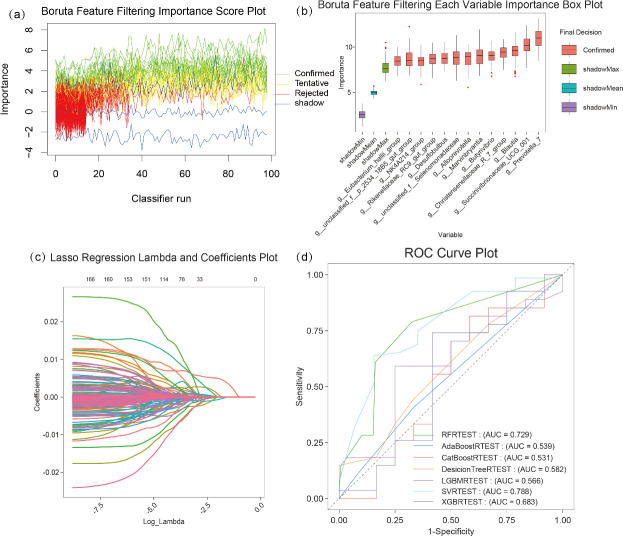
Feature variable selection and prediction model optimization for regression analysis, (**a**) Boruta feature importance score plot, (**b**) Boruta feature importance box plot for individual variables, (**c**) LASSO regression: *λ* parameter versus coefficients plot, and (**d**) ROC curve plot of different models.

In this study, we systematically evaluated the impact of different thresholds on the performance of dairy cow PL predictive models by setting continuous prevalence thresholds (step size = 0.005). Using maximization of *R*² as the criterion, the optimal microbial prevalence threshold was determined to be 0.35% (Fig. S3A). At this threshold, model performance was optimal, yielding 3,589 microbial features (Fig. S3B); *R*² = 0.719 (microbial features accounted for 72% of PL variation), and RMSE = 0.263 (Fig. S3C). Using these 3,589 microbial features, this study further analyzed correlations between the selected taxa and the PL of 341 dairy cows, with individuals divided into training and testing sets at an 8:2 ratio. Boruta and LASSO-Poisson regression identified eight feature microorganisms associated with PL: *g__Prevotella_7*, *g__Succinivibrionaceae*, *g__Blautia*, *g__Christensenellaceae_R_7_group*, *g__Butyrivibrio*, *g__Marvinbryantia*, *g__NK4A214_group*, and *g__Eubacterium_hallii_group* (fig. 5a through c). These features were utilized in seven regression models, yielding AUC values ranging from 0.531 to 0.788. The SVRTEST achieved the highest AUC (0.788), followed by random forest regression (RFRTEST, AUC = 0.729) (Fig. 5d). Evaluation metrics in Table S3 revealed SVRTEST exhibited a coefficient of determination (*R*²) of 0.8033 with minimal prediction-observation discrepancies, establishing it as the optimal model for PL evaluation.

### Development of predictive models for microorganisms associated with dairy cow profitability

**Fig 6:**
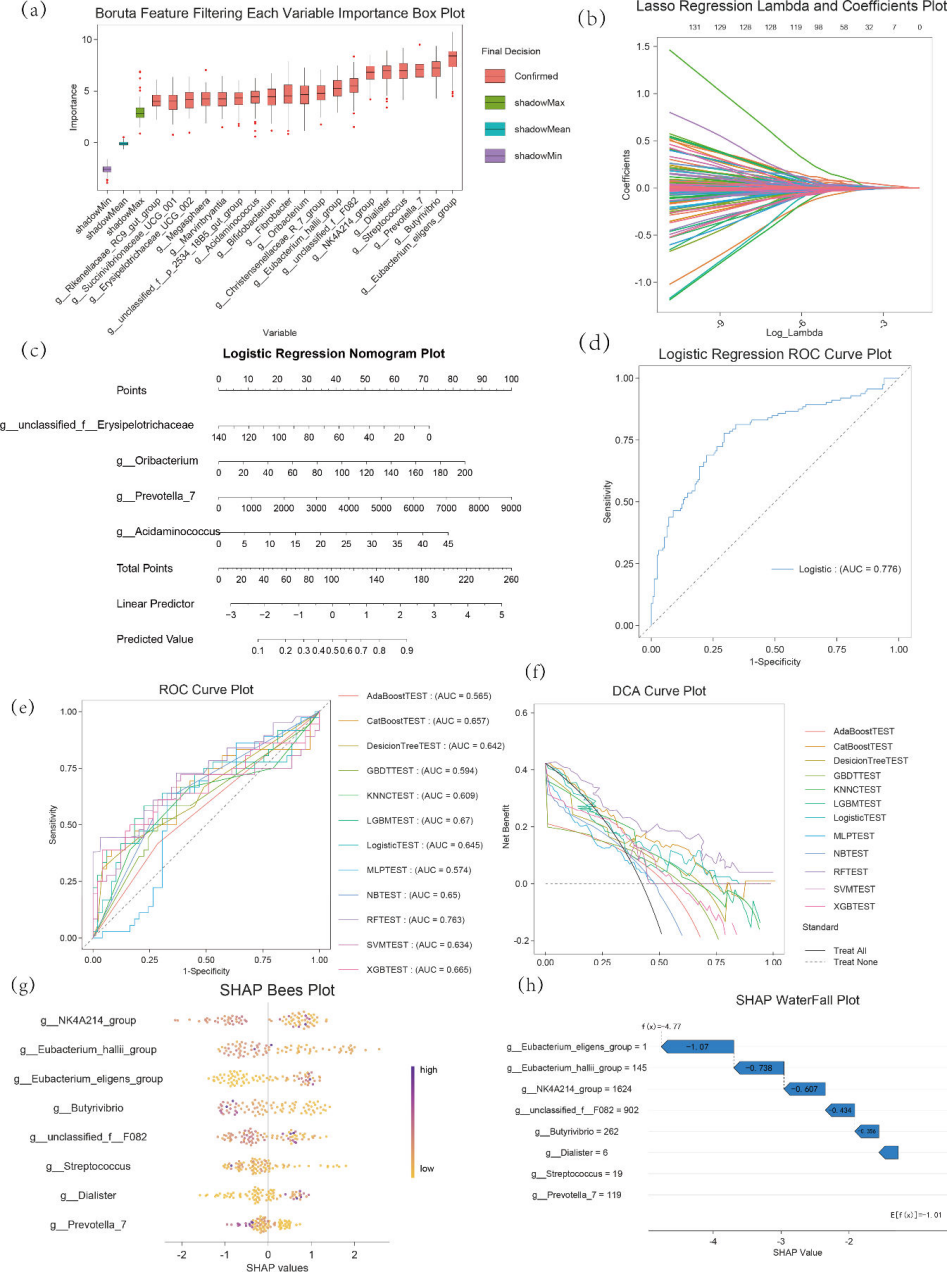
(**a**) Boruta feature filtering each variable important box plot, (**b**) LASSO regression Lambda and coefficients plot, (**c**) Logistic regression Nomogram plot, (**d**) Logistic regression ROC curve plot, (**e**) ROC curve plot of different models, (**f**) DCA curve plot of different models, (**g**) Distribution of SHAP values of each feature on the prediction results in the random forest model (Bees plot), (**h**) Waterfall plot of SHAP value contributions to the prediction result of a single sample in the random forest model; The plot illustrates the SHAP value contributions of each feature to the model's prediction result for a specific sample, starting from the base value (Base Value, the average baseline of model predictions), with the SHAP values of each feature accumulated sequentially.

Rumen microbial fermentation provides 60%–70% of the energy for the host, which is closely associated with milk yield and production performance in dairy cows (20). Predicting the profitability of dairy cow production via rumen microorganisms is critical for the economic planning of dairy farms. Firstly, we performed feature microorganism screening using the Boruta algorithm, identifying 20 feature microorganisms, including *g__Rikenellaceae_RC9_gut_group*, *g__Succinivibrionaceae_UCG_001*, *g__Erysipelotrichaceae_UCG_002*, *g__Megasphaera*, *g__Marvinbryantia*, and *g__Eubacterium_eligens_group* (Fig. 6a and b). Further binomial screening of significant feature microorganisms was conducted using LASSO regression, resulting in four key microorganisms: *g__unclassified_f_Erysipelotrichaceae*, *g__Oribacterium*, *g__Prevotella_7*, and *g__Acidaminococcus* (Fig. 6c). To interpret the predictive capacity and accuracy of these feature variables, we performed logistic regression analysis. The logistic regression model formula is *logit*(*p*) *= −*0.966*-*0.017 *× g__unclassified_f__Erysipelotrichaceae +* 0.014 *× g__Oribacterium +* 0.058 *× g__Acidaminococcus*. A nomogram was used to visualize the contributions and impacts of different microorganisms. The logistic regression model achieved an AUC of 0.776 (Fig. 6d), indicating the predictive potential of these four microorganisms. Combining Boruta and LASSO analyses, *g__NK4A214_group*, *g__Eubacterium_hallii_group*, *g__Eubacterium_eligens_group*, *g__Butyrivibrio*, *g__unclassified_f__F082*, *g__Streptococcus*, *g__Dialister*, and *g__Prevotella_7* were ultimately selected as feature variables. To evaluate their diagnostic performance, we compared 12 classification predictive models, including AdaBoostTEST, CatBoostTEST, Decision TreeTEST, GBDTTEST, KNNCTEST, LGBMTEST, LogisticTEST, MLPTEST, NBTEST, RFTEST, SVMTEST, and XGBTEST. Based on ROC evaluation curves, RFTEST exhibited an AUC of 0.763 (Fig. 6e). Considering the accuracy and F1-Score from the evaluation tables of different models, RFTEST demonstrated the optimal performance (Fig. 6f; Table S4). To better evaluate the reliability of RFTEST, we performed fivefold cross-validation. DCA showed that the net benefit of different subsets was all greater than zero, and the model exhibited high recall and precision, indicating that it is suitable for predictive analysis in this scenario. The distribution of individual microbial feature contributions to RFTEST predictions is shown in (Fig. 6g), where the feature value magnitudes are (Fig. 6) represented by color. *g__NK4A214_group* and *g__Eubacterium_hallii_group* exerted substantial influences on RFTEST results. A Waterfall plot was used to display the cumulative contributions of each feature to the RFTEST model. Starting from a baseline value of *E*[*f*(*X*)] *= −*1.01, each feature adjusted the predicted value negatively based on SHAP predictions. For instance, *g__NK4A214_group* contributed the largest negative adjustment (−1.07), while *g__Butyrivibrio* contributed the smallest (−0.285) (Fig. 6h), suggesting they may play protective roles in anti-aging of dairy cows.

## DISCUSSION

In this study, we first analyzed the correlation between rumen microbiota and PL in 341 Holstein cows across different parities. Our key findings indicate that rumen microbiota vary among cows of different parities, characterized by a decrease in rumen microbial diversity, alterations in the rumen microbial community structure, an increased abundance of pathogenic bacteria within the phylum *Proteobacteria*, and a reduction in the proportions of *Bacteroidota* as the PL of the cow increases. With an extension of PL, microbial community interactions decreased, including reduced interactions between strains and weakened microbial interaction strength. Functional predictions using PICRUSt revealed that the microbiota in cows of the first three parities primarily participate in amino acid metabolism and carbon metabolism, whereas those in cows with five or more parities exhibit extensive nucleotide metabolism and DNA mismatch repair. This may be attributed to microbial metabolic disorders caused by dysbiosis. Subsequently, through machine learning, we identified eight strains of rumen microbiota associated with PL and eight strains linked to ranch economic returns. These results suggest that rumen microbiota was correlated with the PL of cows, and specific rumen microbiota serve as valuable biomarkers with diagnostic significance for both cow PL and ranch profitability.

### Differences in rumen microbiota structure and function across dairy cow parities

Dysbiosis is characterized by an imbalance in the taxonomic composition of the rumen microbiota, with an increase in the abundance of *Proteobacteria* being a common feature of metabolic disorders (21). Our enrichment analysis of rumen microbiota in cows of varying parities revealed that *p__Proteobacteria* was the most significantly enriched microbial phylum in cows with five or more parities. Existing literature supports the association between metabolic disorders and the expansion of *Proteobacteria*. Monocolonization studies conducted in germ-free mice have confirmed the obesity-inducing potential of *Proteobacteria* (22). Unstable gut microbial communities, marked by a high abundance of *Proteobacteria*, may indicate an active feature of metabolic disorders rather than a mere passive result (23). *g__Pseudoramibacter* has been extensively documented as a pathogenic bacterium involved in gingival inflammation (24). *f__Erysipelotrichaceae* is associated with elevated TNF levels and chronic intestinal inflammation in simian immunodeficiency virus-infected animals (25), and it plays a role in redox reactions and pro-inflammatory responses in the body (26). In this study, we found that it was enriched in high-parity cows, potentially linked to metabolic inflammation in these animals. Our comparison of milk production indicated a declining trend in cow productivity with increasing parity. Under consistent dietary and feeding conditions, energy conversion and metabolic efficiency may be the primary factors influencing milk production traits. Combined with the analysis of differential rumen microbial species and microbial functional predictions, the dysbiosis of rumen microbiota in high-parity cows leads to metabolic reprogramming and heightened metabolic inflammatory responses, which may explain the enrichment of pathogenic bacteria such as *p__Proteobacteria*, *g__Pseudoramibacter*, and *g__Erysipelotrichaceae* in these cows. It is important to note that this study employed only microbial diversity sequencing analysis, representing a single methodological approach. Future research should integrate additional omics strategies to further investigate microbial metabolites and host immune status to elucidate their metabolic processes. Furthermore, the microbiota enriched in cows of the first three parities primarily comprises probiotics involved in cellulose degradation, volatile fatty acid production, and amino acid metabolism (20), with *f__Rikenellaceae_RC9_gut_group*, *g__Christensenellaceae_R-7_group*, and *g__Bifidobacterium* being the dominant taxa. Notably, *Eubacterium_hallii_group* has been reported to be associated with glucose utilization and the biosynthesis of fermentation intermediates such as acetate and lactate (27). Therefore, alterations in rumen microbial diversity and community structure may contribute to increased aging and reduced productivity.

### Rumen microbial biomarkers have been identified as predictors of PL and ranch profitability

Liu et al. (28) identified *Prevotellaceae_UCG-003* as a candidate biomarker for distinguishing subacute ruminal acidosis in goats. Through 16S rRNA microbial analysis of fecal samples from patients with diabetic kidney disease (DKD), they found that *g_Escherichia-Shigella* and *g_Prevotella_9* exhibited high discriminatory power in differentiating DKD patients from those with type II diabetes mellitus (29). In this study, we screened eight rumen microbial taxa closely associated with parity using machine learning regression methods. Our observations revealed that the abundance of *Prevotella* spp. was positively correlated with changes in parity, showing less enrichment in high-parity elderly cows. The *Ruminococcaceae NK4A214* group (*P* = 0.0195) was linked to a higher risk of viral hepatitis (30), while *Eubacterium_hallii_group* was associated with an increased risk of Parkinson’s disease (31), and *g__Streptococcus* was related to suppressive responses to immunotherapy (32). Additionally, *Prevotella_7* promotes the development of periodontitis (33). As gram-negative bacteria, *Prevotella* spp*.* are associated with rheumatoid arthritis, ulcerative colitis, and various intestinal diseases (34). Evidently, the microorganisms associated with parity are linked to inflammatory responses and immunosuppression. Furthermore, this study explores the evaluation of ranch profitability using rumen microbiota through 12 machine learning classification methods, identifying eight characteristic microbial taxa. This finding holds significant guiding value for ranch economic planning and adjustments to feeding strategies. In summary, this study provides a biological explanation for the role of microbial communities in the aging process of cows by clarifying the relationship between PLand rumen microbial dysbiosis.

### Conclusion

In this study, we integrated machine learning with traditional bioinformatics to analyze rumen microbiota structure and characteristics across dairy cow parities, revealing for the first time parity-dependent microbial community variations and identifying feature microorganisms associated with PL and farm profitability; however, inherent limitations include reliance on single-omics profiling and lack of experimental validation for feature taxa at the community level. To address these constraints and extend our findings, future research should focus on multi-omics integration to unravel functional mechanisms underlying parity-driven microbial shifts, particularly linking signature microbes to host metabolic pathways such as short-chain fatty acid synthesis and amino acid metabolism, thereby facilitating the translational application of our findings to precision dairy farming.

## Data Availability

The 16S sequencing results have been uploaded to the NCBI database, BioProject ID: PRJNA1375500.

## SUPPLEMENTAL MATERIAL

Supplemental figures spectrum.02969-25-s0001.docx Fig. S1 to S5.

Table S1 spectrum.02969-25-s0002.docx Dairy cow production performance measurement data.

Table S2 spectrum.02969-25-s0003.xlsx Statistics of microbial interactions.

Table S3 spectrum.02969-25-s0004.xlsx Multi-model evaluation node of regression prediction models.

Table S4 spectrum.02969-25-s0005.xlsx Evaluation parameters of regression models.

ASM does not own the copyrights to Supplemental Material that may be linked to, or accessed through, an article. The authors have granted ASM a non-exclusive, world-wide license to publish the Supplemental Material files. Please contact the corresponding author directly for reuse.
