# Supplementary material for: Predicting dairy cattle PL via longitudinal rumen microbiome dynamics using machine learning approaches
Source: Microbiol Spectr. 2026 Apr 8;14(5):e02969-25. doi: 10.1128/spectrum.02969-25 (PMC13141961; doi:10.1128/spectrum.02969-25)
Supplement: Supplemental figures — Fig. S1 to S5. [file spectrum.02969-25-s0001.docx]

**Statistics of milk yield in dairy cows with different parities**


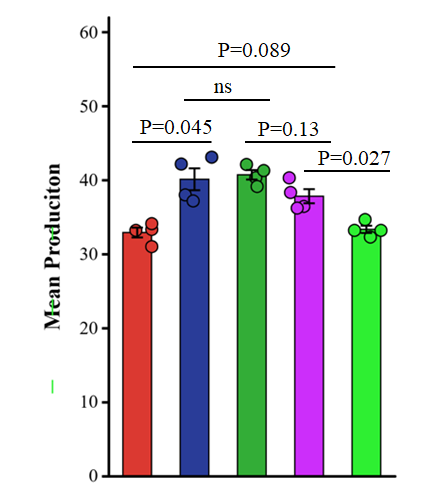

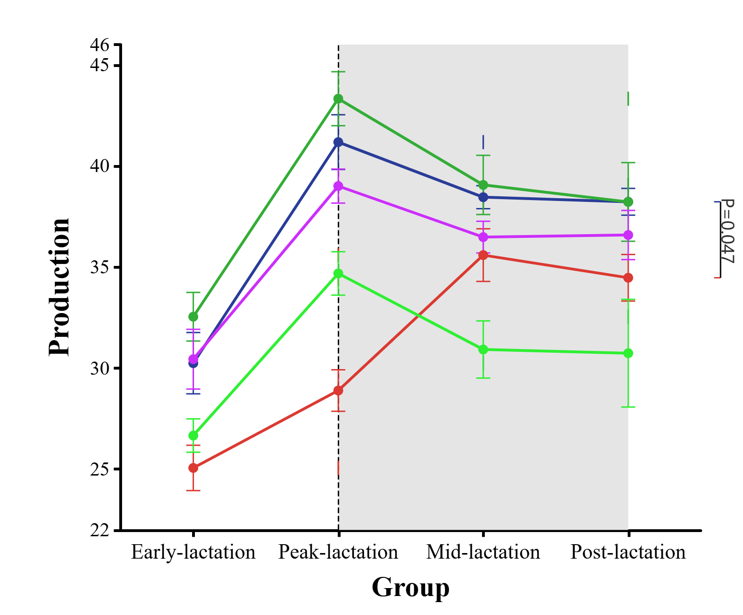

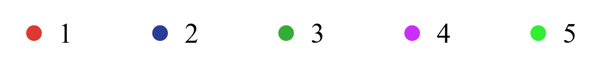

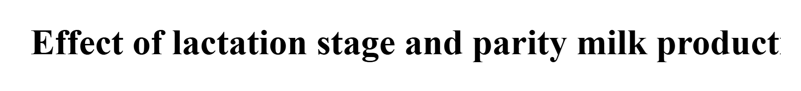


A

B

Fig. S1. A. Distribution of milk yield in dairy cows at different physiological stages; B. Distribution of milk yield in dairy cows with different parities.

**Construction of linear mixed models for milk yield, productive lifespan, and rumen microbial diversity**


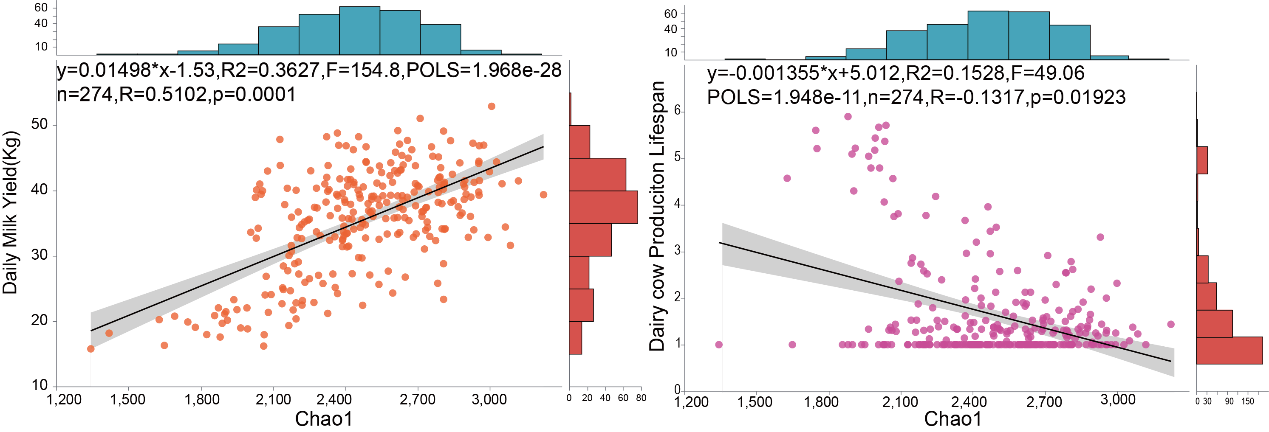


Fig. S2. Correlation between productive lifespan of dairy cows and microbial Chao index

**Microbial threshold**


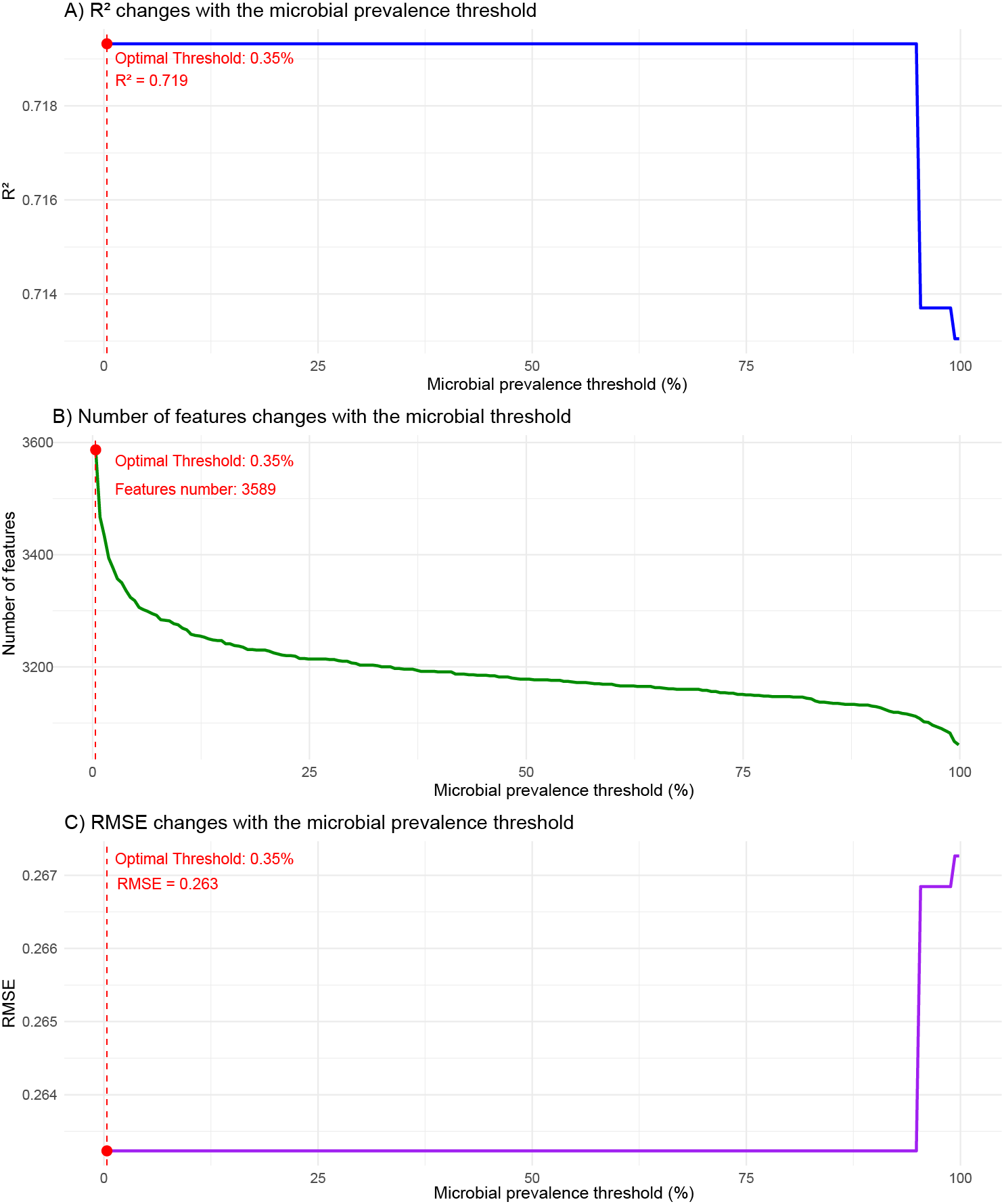


Fig. S3 A) R² trend with threshold: The blue curve represents the relationship between R² and the prevalence threshold, with the red dashed line marking the optimal threshold (0.35%). At this threshold, R² reaches a maximum value of 0.719; B) Number of features trend with threshold: The green curve indicates the negative correlation between the number of features and the prevalence threshold. At the optimal threshold, the number of features is 3589. C). RMSE trend with threshold: The purple curve shows the relationship between RMSE and the prevalence threshold. At the optimal threshold, RMSE reaches a minimum value of 0.263.

**
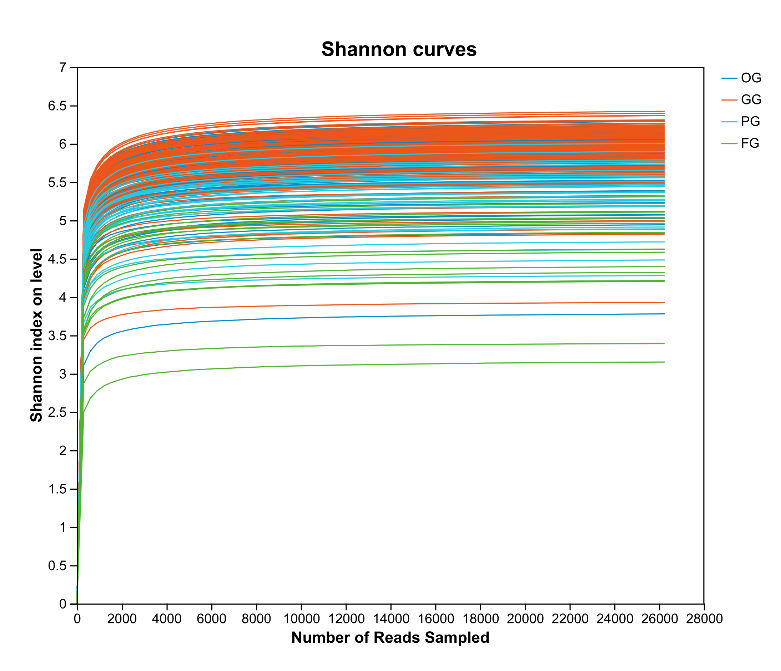
Rarefaction curve**

Fig. S4 Rarefaction curve. For all tested samples, the curves of 16S sequencing depth tend to level off before 4000 reads.

**
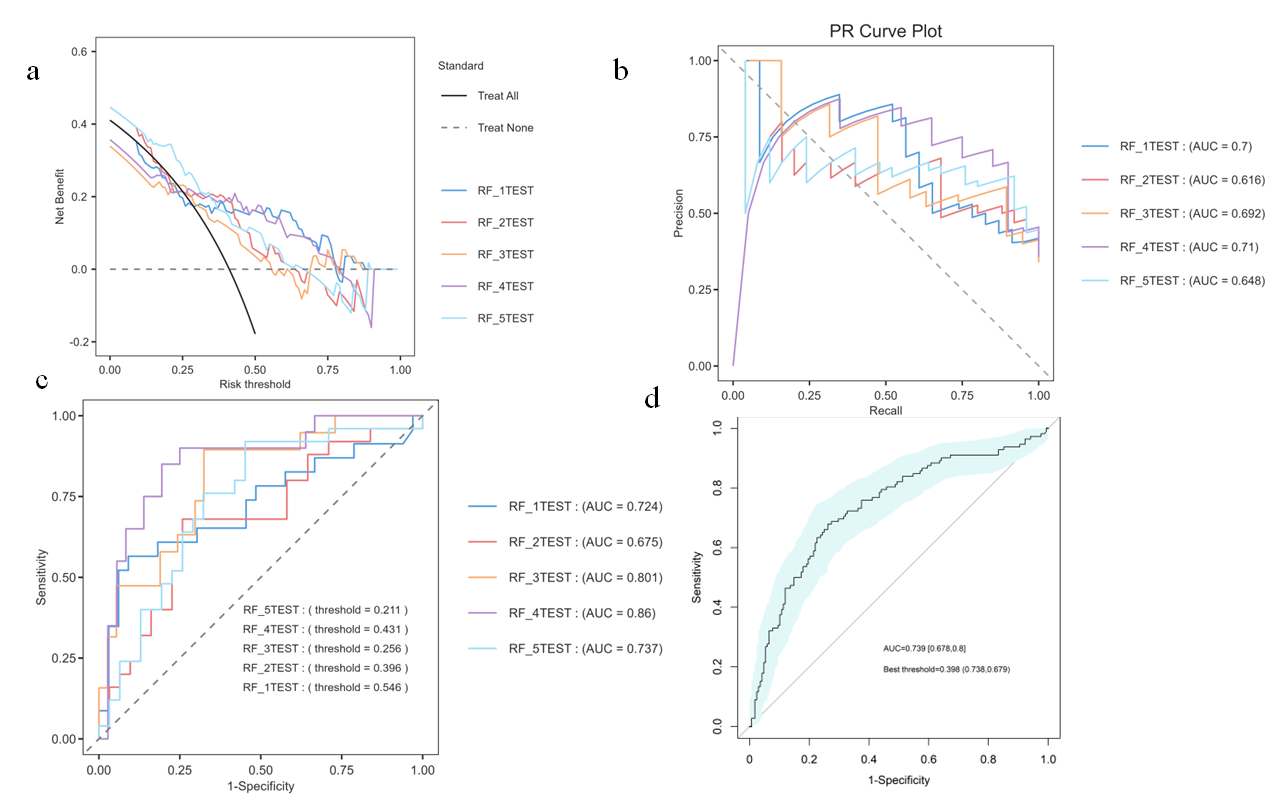
Cross-Validation Analysis of the Random Forest Model**

Fig. S5 a. Decision Curve Analysis (DCA) evaluation curve of the random forest model with five-fold cross-validation. The net benefit of validation sets in different folds was > 0, indicating that the model is meaningful. b. Precision-Recall (PR) curve. As a tool reflecting model performance, we found that the model had high recall and precision, suggesting it is suitable for analysis in this scenario.c, d. ROC evaluation curves of machine learning training results. The AUC values of our ROC curves ranged from 0.67 to 0.81, indicating good reliability of the model.
