# Supplementary material for: Predicting dairy cattle PL via longitudinal rumen microbiome dynamics using machine learning approaches
Source: Microbiol Spectr. 2026 Apr 8;14(5):e02969-25. doi: 10.1128/spectrum.02969-25 (PMC13141961; doi:10.1128/spectrum.02969-25)
Supplement: Table S1 — Dairy cow production performance measurement data. [file spectrum.02969-25-s0002.docx]

**Table S1. Statistical data on production performance of dairy cows with different parities**

| Item | 1^st^ parity | 2^nd^ parity | 3^rd^ parity | 4^th^ parity | 5^th^ and above parity |
| --- | --- | --- | --- | --- | --- |
| Number (entries) | 2068 | 3979 | 1445 | 752 | 587 |
| Daily milk yield（kg） | 33.34±1.09^d^ | 39.39±0.3^a^ | 40.57±0.87^ab^ | 37.10±1.32^c^ | 31.92±0.39^de^ |
| Milk fat percentage（%） | 3.84±0.03^d^ | 3.51±0.15^b^ | 3.48±0.22^c^ | 3.95±0.33^a^ | 3.99±0.69^e^ |
| Milk protein percentage（%） | 3.31±0.19^b^ | 3.22±0.58^a^ | 3.30±0.37^bc^ | 3.42±0.31^a^ | 3.58±0.72^de^ |
| Somatic cell count（×10^4^个/mL） | 10.43±0.04^e^ | 12.35±0.61^cd^ | 13.19±0.29^c^ | 23.97±0.69^b^ | 35.83±1.93^a^ |
| Urea nitrogen（mg/dL） | 15.32±0.09^c^ | 13.34±0.23^e^ | 14.59±0.44^d^ | 16.59±0.41^b^ | 17.83±0.51^a^ |
